# Diffusion Tensor Imaging Studies on Chinese Patients with Social Anxiety Disorder

**DOI:** 10.1155/2014/860658

**Published:** 2014-03-03

**Authors:** Changjian Qiu, Chunyan Zhu, Jingna Zhang, Xiaojing Nie, Yuan Feng, Yajing Meng, Ruizhi Wu, Xiaoqi Huang, Wei Zhang, Qiyong Gong

**Affiliations:** ^1^Mental Health Center, West China Hospital of Sichuan University, Chengdu 610041, China; ^2^The Seventh People's Hospital of Hangzhou, Hangzhou 310013, China; ^3^Key Laboratory for Neuroinformation of Ministry of Education, School of Life Science and Technology, University of Electronic Science and Technology of China, Chengdu 610054, China; ^4^Department of Medical Image, College of Biomedical Engineering, Third Military Medical University, Chongqing 400038, China; ^5^Huaxi MR Research Center (HMRRC), Department of Radiology, West China Hospital of Sichuan University, West China School of Medicine, Chengdu 610041, China

## Abstract

The aim of this study was to explore white-matter disruption in social anxiety disorder (SAD) patients by using diffusion tensor imaging (DTI) and to investigate the relationship between cerebral abnormalities and the severity of the symptoms. Eighteen SAD patients and age- and gender-matched healthy controls were recruited. DTI scans were performed to measure fractional anisotropy (FA) and apparent diffusion coefficient (ADC) for each subject. We used voxel-based analysis to determine the differences of FA and ADC values between the two groups with two-sample *t*-tests. The SAD patient showed significantly decreased FA values in the white matter of the left insula, left inferior frontal gyrus, left middle temporal gyrus, and left inferior parietal gyrus and increased ADC values in the left insula, bilateral inferior frontal gyrus, bilateral middle temporal gyrus, and left inferior parietal gyrus. In SAD patients, we observed a significant negative correlation between FA values in the left insula and the total LSAS scores and a positive correlation between the ADC values in the left inferior frontal gyrus and the total LSAS scores. Above results suggested that white-matter microstructural changes might contribute to the neuropathology of SAD.

## 1. Introduction

Social anxiety disorder (SAD) is a marked and persistent fear of social or performance situations in which the person is exposed to unfamiliar people or to possible scrutiny by others. The situations provoke intense anxiety symptoms in the patients, which are often experienced as somatic symptoms, and, as a result, the individuals may avoid social situations. SAD can be particularly disabling in some patients, leading to reduced likelihood of employment, social isolation, functional disability, and dissatisfaction with life and health [1]. Data obtained by the National Comorbidity Survey indicate that the adult lifetime prevalence of SAD is 13.3% [2]. Recent research also indicated that the 12-month prevalence rate of SAD was 2.48%~7.9% and that the lifetime prevalence was about 3.8%~14.4% [3, 4]. The highly comorbid nature of SAD, which often occurs along with depression, panic disorder, and alcohol abuse, is well established [5, 6].

To date, there is no clear neuroanatomical model for SAD; however, an increasing amount of the neuroscience literature is being devoted to social functioning. The previous functional magnetic resonance imaging (fMRI) studies on SAD found altered brain function in SAD within the medial prefrontal cortex and the limbic regions which formed the corticolimbic circuits, including the amygdala, hippocampus, and insula [7–11]. The amygdala, as a part of the corticolimbic circuits, plays a critical role in learning about environmental predictors of threat and in attention and facial emotions in SAD [12]. Its ability to control fear responses to threatening stimuli was regulated by the hippocampus and medial prefrontal cortex [13]. In addition, the insular cortex is a pivotal structure in the greater limbic lobe and plays a role in diverse functions linked to emotion and memory [14]. However, a better understanding of the neurobiology of SAD would require investigations at the microstructure or anatomical connectivity level, especially in patients not yet exposed to psychotherapy or psychiatric medications.

Diffusion tensor imaging (DTI), which is a recently developed MRI technique, allows for the examination of the integrity of the white-matter microstructure and, thus, serves as an important tool for mapping the anatomical connectivities in humans [15]. DTI measures the directionality and coherence of water diffusion (as reflected by the degree of anisotropy), which provides an estimate of the axonal organization in the brain [16]. The indices used to interpret DTI data were fractional anisotropy (FA) and the apparent diffusion coefficient (ADC). FA values reflect the directionality and coherence of water self-diffusion. Tissues with highly regular fibers have high anisotropy, whereas those with less regular fibers have low anisotropy. Consequently, FA values serve as a quantitative indicator of white-matter coherence or integrity, with lower values signifying decreased structural connectivity in white matter. The ADC values reflect the degree of apparent water diffusivity. Tissues without obstacles (such as cerebrospinal fluid (CSF)) have high water diffusivity, whereas those with obstacles (such as white matter) have low water diffusivity. To date, the use of DTI in the examination of white-matter tracts in SAD patients has been limited. Therefore, the aim of the current study was to explore the differences in the white-matter connectivity in SAD patients and healthy controls by using DTI. In addition, the current study also explored the relation between the severity of SAD symptoms and abnormalities in the white matter.

## 2. Methods and Materials

### 2.1. Subjects

The subjects were 18 adult patients with SAD (according to the DSM-IV criteria) who were recruited from the Outpatient Clinic of the West China Hospital. All subjects were interviewed using the Structured Clinical Interview for DSM-IV criteria Patient Version (SCID-I/P) [17], with additional probes from the self-administered Liebowitz social anxiety scale (LSAS) [18]. All SAD patients were right handed and had LSAS scores ≥38 without psychiatric comorbidities or other medical conditions. None of the patients had received any pharmacological and/or psychotherapeutic treatment. Eighteen healthy controls (HCs), matched for age, sex, handedness, and education, were recruited from the local area by poster advertisement and screened using the SCID-I/P to rule out the presence of current diagnosis or past history of SAD/other axis I psychiatric disorders/axis II antisocial or borderline personality disorders and had LSAS less than 38.

The exclusion criteria for SAD patients and HCs were as follows: (1) any current or past serious medical or neurological illness, including neurologic (Tourette's syndrome, Huntington's disease, Parkinson's disease, encephalitis, stroke, aneurysms, tumors, central nervous system infections, degenerative brain diseases, or trauma), pulmonary, cardiac, renal, hepatic, endocrine, or metabolic (including dehydration) disorders; prior psychosurgery or contraindications to MR scanning, including metal implants, pregnancy, or severe claustrophobia; (2) a current diagnosis or past history of other axis I psychiatric disorders; (3) axis II antisocial or borderline personality disorders (identified using the Structured Clinical Interview for DSM-IV criteria); (4) a history of drug dependence or abuse; (5) a history of psychiatric illness in first-degree relatives.

The study procedure and the involved risks were explained to the subjects; all the subjects gave their written informed consent according to the protocol approved by the ethics committee.

### 2.2. Image Acquisition

All MRI scans were performed using a 3.0 Tesla GE Signa scanner with an eight-channel phased-array head coil. A board-certified neuroradiologist reviewed the scans and found no gross abnormalities in any of the subjects. The DTI was performed using a single-shot echo-planar technique with 15 motion-probing gradient orientations. The key data acquisition parameters for the DTI scan were as follows: TR = 12000 ms; TE = 73.9 ms; flip angle = 90; imaging matrix = 128 × 128; field of view (FOV) = 24 × 24 cm^2^; slice thickness = 3 mm; number of slices = 50; slice gap = 0 mm; number of diffusion gradient directions = 15; *b* = 0 and 1000 s/mm^2^; total scan time = 6 min and 47 s.

### 2.3. Image Processing

The FA and ADC maps were obtained using DTI-Studio (Department of Radiology, Johns Hopkins University School of Medicine, Baltimore, MD, USA; available at http://cmrm.med.jhmi.edu). Image analysis was performed using SPM2 software (developed at the Wellcome Department of Imaging Neuroscience, Institute of Neurology, University College London), which was run on MATLAB7.0 (Mathworks, Sherborn, MA). Spatial normalization is an essential preprocessing step in SPM-based analysis. The contrast settings of the FA and ADC maps differ from those of the T1-weighted and T2-weighted template images provided by SPM2. Thus, the FA and ADC templates specific for this study were created using data from all the participants. Each *b* = 0 image in the native space was standardized using the T2 template supplied with the SPM2 software, and the normalization parameter was applied to the respective *b* = 0, FA, and ADC maps. The normalized maps were smoothed with a 6 mm full width at half-maximum (FWHM) isotropic Gaussian kernel, and the mean images (*b* = 0 template, FA template, and ADC template) were created. Then, all the FA and ADC maps in native space were transformed into stereotactic space by registering each image with the customized FA and ADC templates.

### 2.4. Statistical Analysis

Differences between the demographic variables of the 2 groups were examined using independent-group *t*-tests. Two-sample *t*-tests were performed for each voxel of the FA and ADC values across the entire brain. In these analyses, the statistical threshold was defined as a *t* value above 2.44 (*P* < 0.01, uncorrected). We used MarsBar (http://marsbar.sourceforge.net) for extracting the FA and ADC values in the ROIs (region of interest) by two steps: firstly, ROIs were defined as regions in which the FA and ADC values of the SAD patients were abnormal from two-sample *t*-tests with a cluster size bigger than 50; secondly, values were extracted from FA or ADC map of each SAD patient separately and investigated the correlations between ROIs' FA and ADC values and the score of LSAS in the SAD patients. Correlations were calculated using Pearson's correlation analysis, and a *P* value less than 0.05 was considered significant.

## 3. Results

**Figure 1:**
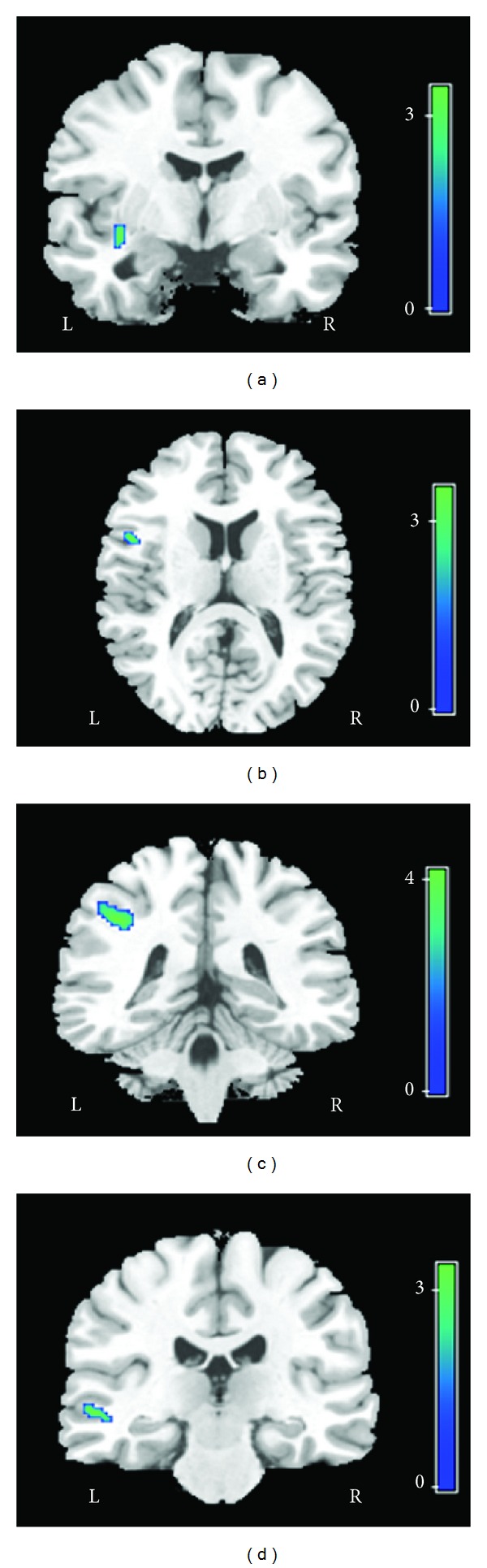
Four clusters of significantly decreased FA values in the left insula cortex (a), left inferior frontal gyrus (b), left inferior parietal gyrus (c), and left middle temporal gyrus (d) in the SAD patient group, in comparison to the corresponding values of the HCs. The clusters are superimposed onto the images from a representative T1-weighted MRI study in Montreal Neurological Institute (MNI) space (Courtesy of MRIcro, Chris Rorden).

**Figure 2:**
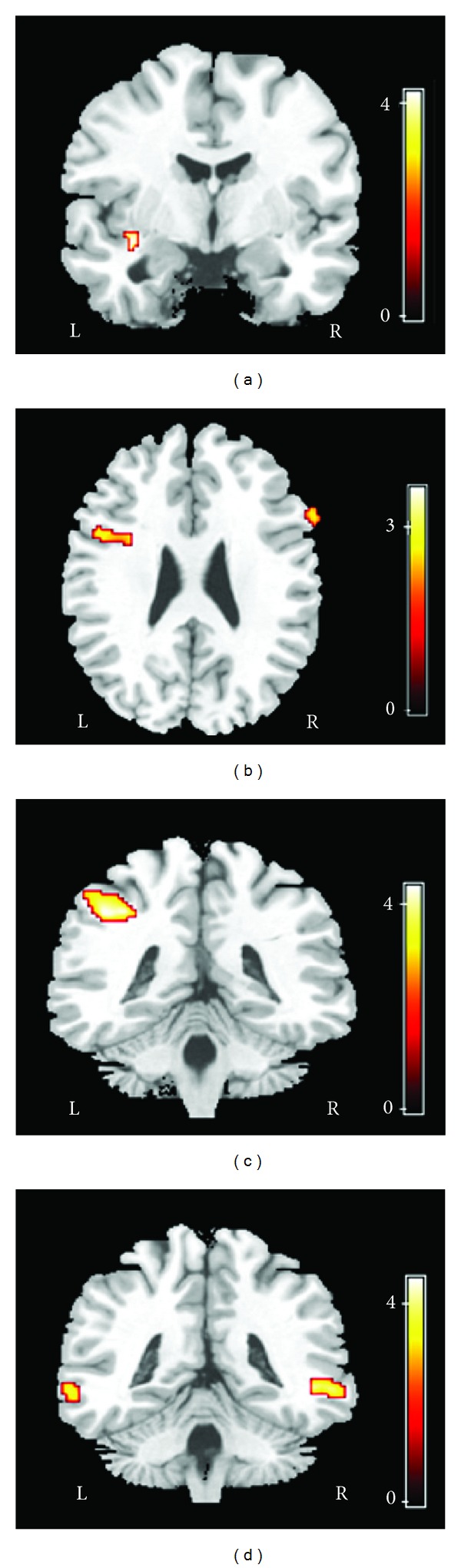
Six clusters of significantly increased ADC values in the left insula (a), left and right inferior frontal gyrus (b), left inferior parietal gyrus (c), and left and right middle temporal gyrus (d) of the SAD patient group, in comparison to the corresponding values of the HCs. The clusters are superimposed on the images from a representative T1-weighted MRI study in MNI space (Courtesy of MRIcro, Chris Rorden).

**Figure 3:**
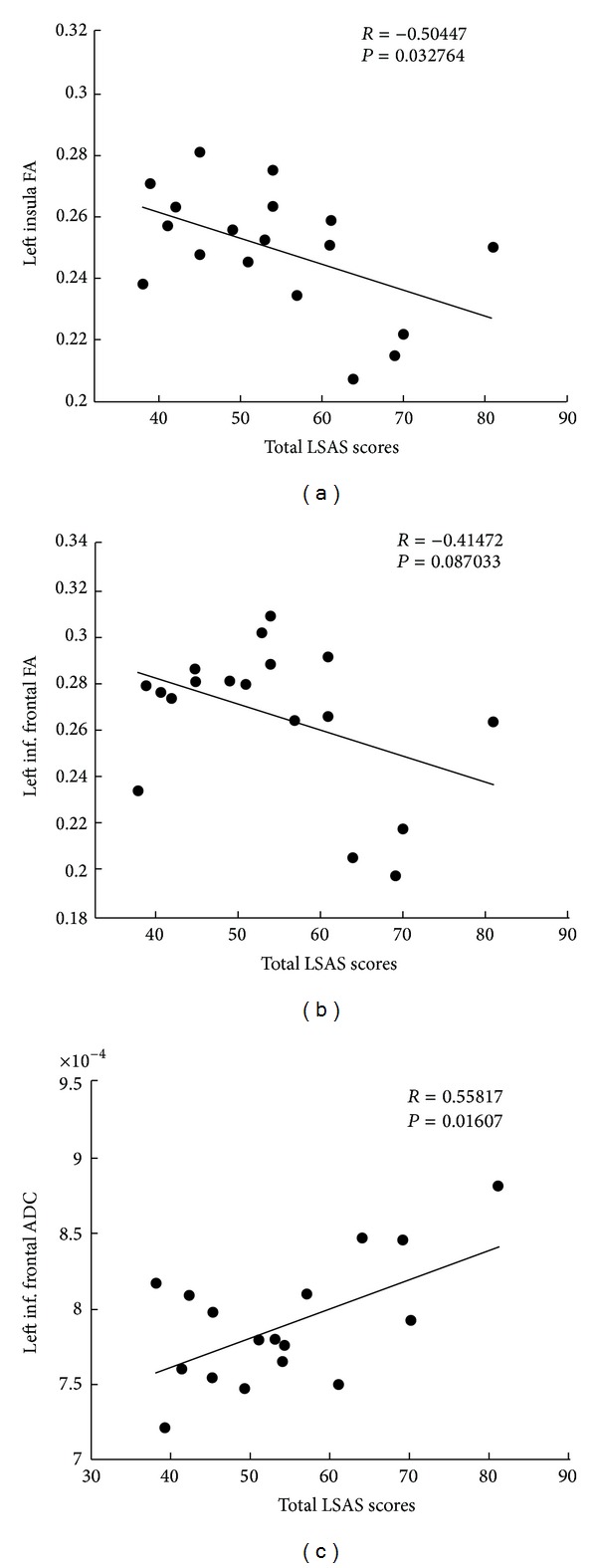
(a) A cluster in the left insula region with a significant negative correlation between the FA values and the total LSAS scores. (b) A cluster in the left inferior frontal region with a trend of a negative correlation between the FA values and total LSAS scores. (c) A cluster in the left inferior frontal region with a significant positive correlation between the ADC values and the total LSAS scores.

Age, sex, handedness, and education did not differ significantly between the SAD patients and the HCs (Table 1). In comparison to the HCs, the SAD patients had decreased FA values in the white matter of the left insula, left inferior frontal gyrus, left middle temporal gyrus, and left inferior parietal gyrus (Figure 1; Table 2). The SAD patients also showed increased ADC values in the left and right inferior frontal gyrus, left and right middle temporal gyrus, left inferior parietal gyrus, and left insula (Figure 2; Table 2). In addition, we found a negative correlation between the decreased FA values in the left insula and the total LSAS scores of the SAD patients (*r* = −0.504, *P* = 0.033) and a trend of negative correlation between FA values and the left inferior frontal gyrus (*r* = −0.414, *P* = 0.087); there was a positive correlation between the increased ADC values in the left inferior frontal gyrus and the total LSAS scores of the SAD patients (*r* = 0.558, *P* = 0.016) (Figure 3).

## 4. Discussion

Few studies have examined the brain white matter in SAD patients. The preliminary findings provided evidence of abnormal white-matter microstructure in SAD patients, as inferred from the DTI results. Specifically, the findings of this study were as follows: (1) the SAD patients showed decreased FA values in the left insula, left inferior frontal gyrus, left middle temporal gyrus, and left inferior parietal gyrus and a negative correlation between the decreased FA values in the left insula and the total LSAS scores; (2) the SAD patients also showed increased ADC values in the left insula, left and right inferior frontal gyrus, left and right middle temporal gyrus, and left inferior parietal gyrus and a positive correlation between the increased ADC values in the left inferior frontal gyrus and the total LSAS scores.

These findings corroborate the findings of prior studies, which reported functional abnormalities in the corticolimbic circuits of SAD patients [8–10]. In this study, significant white-matter abnormalities were observed on both sides of the frontal cortex of the subjects with SAD. A recent study on SAD conducted using social anxiety imagery condition showed several regions of altered regional cerebral blood flow (rCBF)/activation [19]. Changes in rCBF/activation were also noted following pharmacotherapy. A recent PET study showed that the anticipatory anxiety in SAD subjects was associated with decreased bilateral frontal activation [20]. Another recent SAD study revealed a significant positive correlation between the resting perfusion values and the total LSAS scores at the left frontal cortex [21]. A review of the role of the medial frontal cortex (MFC) in social cognition by Amodio and Frith [22] suggests that the anterior rostral MFC plays a role in social cognition by integrating the afferents from the posterior rostral MFC (involved in the monitoring of action) and the orbitofrontal cortex (involved in the monitoring of reward or punishment). The medial prefrontal cortex is hypothesized to play a role in the inhibition or extinction of excessive corticolimbic activity in patients with anxiety disorders [23]. If this is the case, then frontal findings from this study may indicate dysfunction in the cortical regions of the SAD patients, which contribute directly to the etiology of SAD.

The insula is associated with strong emotional responses, such as disgust [24, 25] as well as the representation of visceral sensation [26], and might play a role in several anxiety disorders [27]. In this study, the FA and ADC values in the left insula of the SAD patients were significantly lower and higher, respectively, than the corresponding values in the HCs. In addition, this result was also strengthened by the independent finding of a correlation between the total LSAS score and FA value in the SAD group. Insula activation results for SAD patients were found to be different from those for the HCs, which paralleled the results of previous studies [10, 28]. Interestingly, there is evidence that brain activation in response to threatening faces in SAD patients differs greatly from the activation in HCs when facial emotional expressions are task irrelevant, which suggests an automatic processing of facial anger cues in SAD patients [10]. The functional and structural abnormalities in SAD patients may affect their social cognitive processing circuits. We speculated that insula abnormalities were one of the neurobiological mechanisms. In addition, a recent study found that if the anterior insula participated in anticipatory processing [29], then abnormalities in the white matter of the insula may be interpreted as neural mechanism for symptom of anticipatory anxiety in SAD patients.

Our study reported abnormalities in the white matter of the middle temporal gyrus and inferior parietal gyrus in subjects with SAD. Many studies have revealed that, in the temporal cortex of monkeys and humans, the temporoparietal junction, which is located primarily in the superior temporal sulcus (STS) region, is activated by movements of the eyes, mouth, hands, and body, suggesting that this junction is involved in the analysis of biological motion [25, 30]. STS, the core system perceptual area, has been associated with the perception of expression [31, 32], the evaluation of the intentions and personality traits of others [32, 33], and, more generally, the social evaluation of others [34]. Face recognition and analysis of facial expression form an important part of everyday interactions among humans. Previous studies have suggested that SAD patients show biased processing of emotional expressions and personality traits, resulting in the fear of social interaction [35, 36]. A significant behavioral effect while processing socially relevant stimuli, face processing in particular, has been shown in previous studies. Specifically, behavioral studies have reported that SAD patients tend to judge neutral faces negatively [37], remember critical faces better than accepting ones [38], and scan faces with a different pattern of eye movements than that used by the HCs [36]. These results suggest that SAD view still images of faces with a negative or wary attitude. Straube et al. found that in a comparison of angry versus neutral faces in an implicit task, activation of the STS region in the SAD patients was stronger than that in the HCs [10]. These results suggest a specific pattern of activity in the different parts of the distributed neural system for face perception in SAD patients. Abnormalities in the above-mentioned neuroanatomical regions may supply the neurobiological basis for biased processing of information in SAD patients.

It is particularly interesting that all of significantly decreased FA regions occurred in the left hemisphere including the left insula cortex, left inferior frontal gyrus, left inferior parietal gyrus, and left middle temporal gyrus in the SAD patient group. The possible lateralization of emotion was found in brain structures, such as the frontal cortex, the amygdala, and insular cortex in a previous meta-analysis of neuroimaging studies [39]. Our results were also consistent with recent functional neuroimaging studies showing an overall lateralization of cortex and limbic system activations to the left hemisphere, particularly for corticolimbic circuits [40]. Further studies specifically designed to examine whether the lateralization of the function and structure exists and how it works in SAD patients would be helpful to clarify the disease etiology.

The current study had a number of limitations. Firstly, the patient group was interviewed using the Structured Clinical Interview for the Diagnosis of Axis-I Disorders, but they were not classified into general SAD or specific SAD groups. Secondly, while the 2 groups of subjects were comparable with respect to age, gender, and level of education, the comparability of the 2 groups for other potentially confounding factors, such as socioeconomic status, was not assessed. Finally, it needs to be noted that this study used a low significance threshold for a whole-brain, voxel-wise analysis (*P* < 0.01 uncorrected). Although this situation was not ideal, we believed that we were still able to perform a comparison of 2 reasonably well-defined groups. The emphasis in this analysis was to maximize sensitivity and to use a less strict threshold so as to avoid overlooking significant findings. Future studies with larger sample size may help to see whether there would be more robust result for SAD patients concerning white-matter abnormalities. Despite these limitations, we believe that our findings will contribute to the growing literature on the imaging studies of anxiety disorders.

## 5. Conclusion

This study showed several brain-lobe abnormalities in the white matter of the SAD patient group. These findings were parallel to those of previous studies that showed functional abnormalities in these regions; the findings were also consistent with the hypothesized role of these regions in the modulation of excessive limbic activity in anxiety disorders. The left insula and the left inferior frontal gyrus in SAD patients showed a significant correlation between the total LSAS score and the FA or ADC values, which may point to the defective perception of self and others in these patients. Future studies with tractography would provide more information referring to the disruption of corticolimbic circuits in SAD patients.

## Figures and Tables

**Table 1:** Demographic characteristics (mean ± SD) of subjects.

	SAD (*N* = 18)	HC (*N* = 18)	*t* (df = 34)	*P*
Age	22.72 ± 3.85	21.78 ± 3.90	0.73	0.47
Gender	12 M/6 F	12 M/6 F	—	—
Education	14.11 ± 1.53	14.05 ± 2.04	0.09	0.927
Duration	49.22 ± 40.17			
Total LSAS	54.11 ± 11.90	19.50 ± 8.50	10.04	0.000

SAD: social anxiety disorder; HC: healthy control; M: male; F: female.

Educational background was measured according to the years of education.

The severity of social anxiety was measured using the Liebowitz social anxiety scale.

Duration: the time from the beginning of the first episode to the time of assessment (measured in months).

**Table 2:** Differences between the FA and ADC values of the SAD patients and HCs.

Anatomical region	Talairach coordinates at the center of the cluster			Cluster sizes	*t* value
	*x*	*y*	*z*		
Lower FA					
Inferior frontal gyrus (L)	−51	10	12	66	3.5212
Insula (L)	−36	4	−5	114	3.4206
Inferior parietal gyrus (L)	−42	−35	39	226	4.1552
Middle temporal gyrus (L)	−57	−23	−2	164	3.3456
Higher ADC					
Inferior frontal gyrus (L)	−50	11	18	173	4.8080
Inferior frontal gyrus (R)	59	21	27	81	3.6572
Insula (L)	−38	−4	−10	78	3.1251
Inferior parietal gyrus (L)	−44	−37	42	382	4.3239
Middle temporal gyrus (L)	−40	3	−24	443	4.4674
Middle temporal gyrus (R)	50	−37	−2	758	4.5024
